# VORFFIP-Driven Dock: V-D^2^OCK, a Fast and Accurate Protein Docking Strategy

**DOI:** 10.1371/journal.pone.0118107

**Published:** 2015-03-12

**Authors:** Joan Segura, Manuel Alejandro Marín-López, Pamela F. Jones, Baldo Oliva, Narcis Fernandez-Fuentes

**Affiliations:** 1 Leeds Institute of Molecular Medicine, School of Medicine, University of Leeds, Leeds, LS9 7TF, United Kingdom; 2 Structural Bioinformatics Lab (GRIB-IMIM), Department of Experimental and Health Sciences, Universitat Pompeu Fabra, 08003 Barcelona, Catalonia, Spain; Koç University, TURKEY

## Abstract

The experimental determination of the structure of protein complexes cannot keep pace with the generation of interactomic data, hence resulting in an ever-expanding gap. As the structural details of protein complexes are central to a full understanding of the function and dynamics of the cell machinery, alternative strategies are needed to circumvent the bottleneck in structure determination. Computational protein docking is a valid and valuable approach to model the structure of protein complexes. In this work, we describe a novel computational strategy to predict the structure of protein complexes based on data-driven docking: VORFFIP-driven dock (V-D^2^OCK). This new approach makes use of our newly described method to predict functional sites in protein structures, VORFFIP, to define the region to be sampled during docking and structural clustering to reduce the number of models to be examined by users. V-D^2^OCK has been benchmarked using a validated and diverse set of protein complexes and compared to a state-of-art docking method. The speed and accuracy compared to contemporary tools justifies the potential use of VD^2^OCK for high-throughput, genome-wide, protein docking. Finally, we have developed a web interface that allows users to browser and visualize V-D^2^OCK predictions from the convenience of their web-browsers.

## Introduction

One of the most prevalent challenges in the post-genomic era is the charting and description of the protein networks that underpin cellular functions. Large-scale interactomic experiments (e.g.[1,2]) sought to describe the protein interactions that occur in cells, and albeit valuable, most of the information derived from these experiments does not provide the underlying structural, atomic details of the interactions. These details are central in order to realize the full potential of interactomic data in rational approaches such as the development of novel drugs to target protein interfaces[3] or understanding the effect of mutations[4], for example. Computational methods can be used to derive structural models of protein complexes (reviewed in [5] and references therein), which can then be used as the starting point for further research approaches.

Protein docking represents one such computational approach. Protein docking is an active field of research; shown by the number of participants in the regular Critical Assessment of PRediction of Interactions (CAPRI) exercises[6] and the number of publications devoted to the field. Protein docking methods can be broadly divided in two groups: unbiased (or *ab initio*) and biased (or data–driven) approaches and implementations of both have been described in the scientific literature (e.g. [7–18]). The major difference between *ab initio* and data-driven docking is that the latter group restricts the sampling of docking to selected region(s) of the proteins, whereas in the former group the sampling of the docking space is not restricted. The constraints to guide data-driven docking can be derived from either experimental methods (e.g. Hydrogen-Deuterium exchange data[19]) or computational predictions (e.g. binding site predictions [8]).

In this work, we present the development of a high-throughput computational docking strategy: V-D^2^OCK, which combines protein-binding site prediction and data-driven docking. V-D^2^OCK also includes a clustering step to reduce the number of docking poses while preserving the conformational richness of the sampling. Our results show that V-D^2^OCK is a competitive and faster approach than *ab initio* docking and successfully samples the docking space generating near-native docking poses. The clustering step resulted in only limited decrease in performance while substantially reducing the number of docking solutions, a desirable characteristic in a day-to-day use of this technology. V-D^2^OCK is accessible as a web application at http://www.bioinsilico.org/VD2OCK. The web-server includes a bespoke and interactive graphic viewer that allows users to examine and manipulate the docking poses using the web-browser.

## Material and Methods

### Datasets

The benchmarking of V-D^2^OCK was performed using Benchmark v4.0 [20] referred here as the B04 set. B04 was specifically compiled to test docking methods and it consists of 176 complexes classified in: rigid-body, medium difficulty and difficult cases depending on the structural changes upon complex formation. The atomic structures for the proteins are available in both bound and unbound conformations.

In the case of the V-PATCH algorithm, a dataset referred to here as SOB4 was derived from an original set of protein complexes described in Ofran et al.[21] after removing any protein complexes whose SCOP superfamily [22] was represented in B04. This set was used to train VORFFIP [23], hence avoiding any bias between the training and testing set. The protein interfaces of the native complexes in B04 were determined using DIMPLOT [24] on the bound complexes. The binding site prediction scores were computed using VORFFIP on the unbound structures.

### V-D^2^OCK algorithm

**Fig 1:**
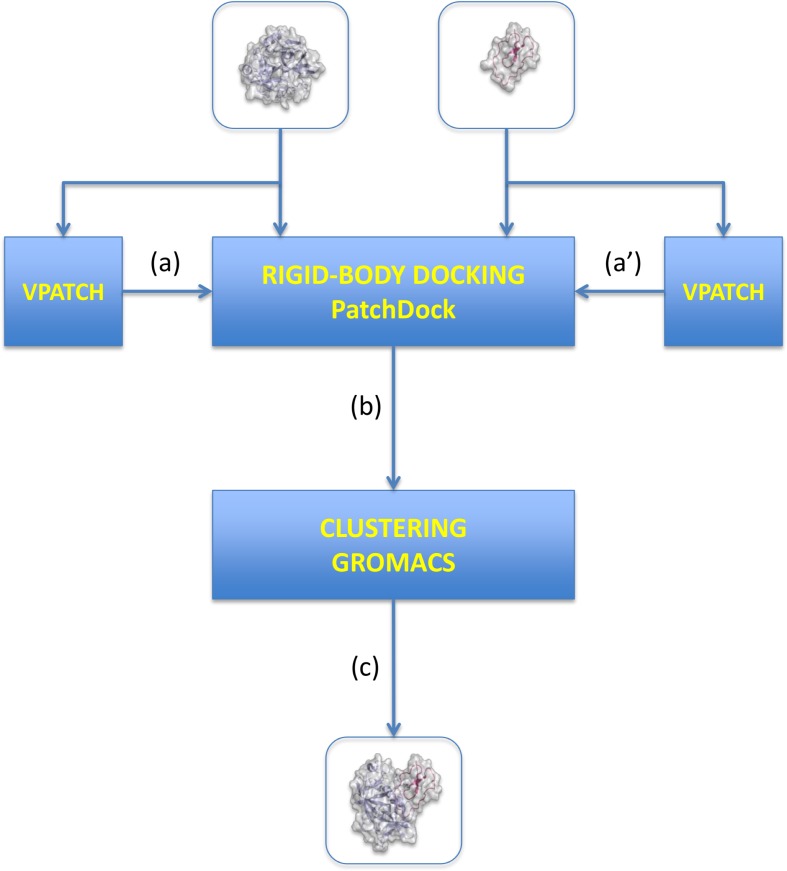
VD^2^OCK workflow. (a) (a’) V-PATCH algorithm is used to define the protein binding sites based; (b) rigid-body docking is driven by interface predictions; (and c) clustering stage where dockings poses are structurally clustered and clusters’ centroids selected as representatives.

The V-D^2^OCK algorithm is composed of different steps that include the prediction of binding sites in proteins, sampling of the docking space using data-driven docking and the clustering of the docking poses to reduce the number examined (Fig. 1).

### From single residues to interaction patches: V-PATCH

The first step of the algorithm involves the delineation of the binding sites in both partners. VORFFIP [23] was used to assign scores to individual scores using the unbound structure that were then used to compute the interface patches by V-PATCH. VORFFIP scores are fed into V-PATCH to define explicit binding sites in a protein structure by an automatic and iterative clustering of residues that include: (i) the initial patch generation; (ii) patch selection; and (iii) patch extension.

In the initial patch generation, a new score named extended score: $s_{i}^{*}$ (1) is calculated for each residue that includes VORFFIP’s original score and the contribution of the environment scores as defined in our earlier work [23]. Let {(*a*_*k*_,*s*_*k*_);*k* = 1,…,*N*} be the residues and predicted scores of a given protein and {a_*j*_; j = 1, …,n} neighbours of residue *a*_*i*_, then $s_{i}^{*}$ is defined as

$$s_{i}^{*}=0.5\left[s_{j}^{'}+\sum_{j=1}^{n}c_{ij}s_{j}^{'}\right]$$ (1)

where *c*_*ij*_ is the contact strength between *a*_*i*_ and *a*_*j*_ and $s_{j}^{'}$ (2) is the normalized score calculated as

$$s_{j}^{'}=\frac{s_{j}-m}{M-m}$$ (2)

being *m* = min{*s*_*i*_; *i* = 1, …,*N*} and *M* = max{*s*_*i*_; *i* = 1,…,*N*}.

The initial patches are then started with the residues with the highest scores and extended to any neighbouring residues until $s_{i}^{*}$ falls below a threshold *α* or hard-average cut off. The parameter *α* was calculated using the average of the extended scores for interface residues in the complexes of SOB4 dataset.

During the patch selection stage, redundant patches are removed. The list of patches is sorted by size, and any smaller patches that are also associated with a larger patch are removed, retaining only the largest patch. The last stage of the algorithm extends the patches to maximize the size of the interface patch by including neighbouring residues that were not selected in the previous rounds and whose extended score is above a certain threshold *β*. The parameter *β*, named the soft average cut-off, is calculated by computing the average of extended scores in the case of residues that are not part of protein interfaces in SOB4 dataset. An explicit pseudo code implementation of the algorithm is available in the supplementary material (S1 File).

### Data-driven docking and clustering of the docking space

The patches computed by V-PATCH are then used to guide the docking of protein partners. V-D^2^OCK utilizes PatchDock[25] [15] to perform the docking of the proteins. The list of residues conforming the patches identified by V-PATCH is given as an input to PatchDock.

The third stage of the algorithm is the structural clustering of the docking poses to reduce the redundancy and size. This method used the g_cluster application, part of the GROMACS package [26]. The g_cluster is executed with default parameters except for the RMSD cut-off, which is set up to 5 Angstroms (Ang), based on the threshold used in the CAPRI competition [6] to define a docking solution as medium accuracy. Thus, this ensures that all members within a cluster will have a similar RMSD if compared to the centroid.

### Scoring of docking models

Three different scoring functions were used to rank the docking models: (i) PatchDock native score[15]; (ii) the ES3DC potential, a distance and environment dependent knowledge-based statistical potential[27]; and (iii) ZRANK[28]. The complete set of docking complexes derived for the entire B04 using V-D^2^OCK is available as a compressed file (bzip2) at http://www.bioinsilico.org/VD2OCK/PD_B4_results.tar.bz2, upon request to the authors or at the Harvard Dataverse Network (S2 File).

### Statistical measures

Four widely used statistical measures were used to assess the performance: Recall (1), Precision (2), the Matthews Correlation Coefficient (MCC)(3), and the F1 score(4). Formally,

$$\mathrm{Recall}=\frac{TP}{TP+FN}$$ (1)

$$\mathrm{Precision}=\frac{TP}{TP+FP}$$ (2)

$$\mathrm{MCC}=\frac{TP\times TN-FP\times FN}{\sqrt{\left(TP+FN\right)\left(TP+FP\right)\left(TN+FP\right)\left(TN+FN\right)}}$$ (3)

$$F1=\frac{2TP}{2TP+FN+FP}$$ (4)

where TP is the number of true positives, TN true negatives, FP false positives, and FN the number of false negatives.

## Results and Discussion

### V-Patch algorithm

The V-PATCH algorithm was designed to define interface patches based on VORFFIP predictions[23]. The patches defined by V-PATCH were compared to the native interfaces of the protein complexes in the dataset B04. In 28 cases out 175 complexes, the predicted interface matched 80% or more of the native interface residues, in 82 cases the success ranged between 20 and 80% overlap and in 12 cases the overlap between the native and predicted interface was less than 5%. Having a high overlap between the predicted and native interface is highly desirable in data-driven docking, albeit not vital, since including only a few native contacts (i.e. low overlap) is usually enough and limitations can be corrected during the docking process as recently discussed [8]. On the other hand, over-predicting also presents disadvantages: it increases of the search space and hence computational time and the number of docking solutions to rank is higher.

To fully assess the advantages of V-PATCH algorithm, the accuracy of predicted interface patches defined on the basis of a fixed threshold (both raw and normalized VORFFIP scores) and V-PATCH were compared. As shown in Table 1, V-PATCH performs better than fixed threshold for all the statistical measures: recall, precision, F1 scores and MCC. V-PATCH has the clear advantage that no thresholds need to be defined. Moreover, V-PATCH has been designed such that multiple, independent, patches on the surface can be defined, i.e. it can generate different, independent, binding sites.

**Table 1:** Statistical performance of V-PATCH and fixed thresholds.

Method	R(%)	P(%)	F1	MCC
**V-PATCH**	61	27	0.37	0.34
**Fixed Threshold (raw)**	60	22	0.32	0.29
**Fixed Threshold (norm)**	60	24	0.34	0.30

VPATCH and fixed thresholds using raw and normalized score were used to compare performance. Results are shown for (R) recall, (P) precision, the (F1) F1 score and (MCC) Matthews correlation coefficient

### Sampling of docking space on a validated set: B04

The first question to address was the completeness of sampling of the docking space by V-D^2^OCK in order to understand whether near-native structural poses were generated. The performance of sampling was assessed in terms of the ligand-Root Mean Square Deviation, l-RMSD, adapting the scoring scheme from CAPRI[6]: high accuracy (three-stars), medium accuracy (two-stars), acceptable (one-star) or wrong. With an average number of around 4000 docking poses per complex, V-D^2^OCK yielded acceptable and medium quality structural models for over 70% of the cases; one case ranked as high-quality (the Falcipain-2 and Cystatin complex [PDB code 1yvb]) and in 30% of the cases the docking failed to sample any suitable conformation (see Table 2). Specific information on each individual protein including the theoretical minimum RMSD, i.e. best docking pose, is shown in S1 Table (supplementary material).

**Table 2:** Effect of clustering in the quality of the models.

# of solutions to cluster(a)	CAPRI evaluation system(b)				# Docking poses(c)
	A	B	C	D	
**No clustering**	1	46	75	53	4509
**All clusters**	0	21	93	61	898
**1000**	0	20	83	72	635
**200**	0	17	73	85	218
**100**	0	13	60	102	162
**50**	0	9	47	119	96

(a) Sets of solutions used: all poses, centroids for all clusters, centroids for the top 1000, centroids for the top 200, centroids for the top 100 and centroids for the top 50 clusters respectively. (b) CAPRI evaluation system where A, B, C and D represent the number of predictions considered as high-quality quality (three stars), medium quality (two stars), acceptable and wrong respectively are shown alongside the average number of docking models (c)

V-D^2^OCK generates an average of 1353 docking poses per interface and 4509 docking poses per complex. Given the large number of docking poses and the challenges it might present for routine use and downstream processing such as energy minimization, a clustering step based on structural similarity was devised. Different clustering cut-offs were explored to assess the impact on the quality of the sampling. The l-RMSD of the best docking poses was computed when: (i) considering all poses (no clustering), (ii) considering the centroids of all clusters; (iii) considering the centroids of the top 1000 clusters; (iv) considering the centroids of the top 200 clusters; (v) considering the centroids of the top 100 clusters; and (vi) considering the centroids of top 50 clusters as per PatchDock scoring function[25]. As shown in Table 2, increasing in clustering stringency results in a decrease in the quality of the models. This is due to the intrinsic structural variability among the models that belong to the same cluster as only the centroid is considered for calculation purposes. However, there is a clear advantage in the clustering as the number of poses reduces dramatically, thus reducing the number of models to be inspected, while the reduction in the quality of the models is lesser to some extend in comparison (e.g. no clustering vs. all poses). Moreover, all members of the cluster can be easily retrieved upon inspection of the structure of the centroid (see V-D^2^OCK web server).

**Fig 2:**
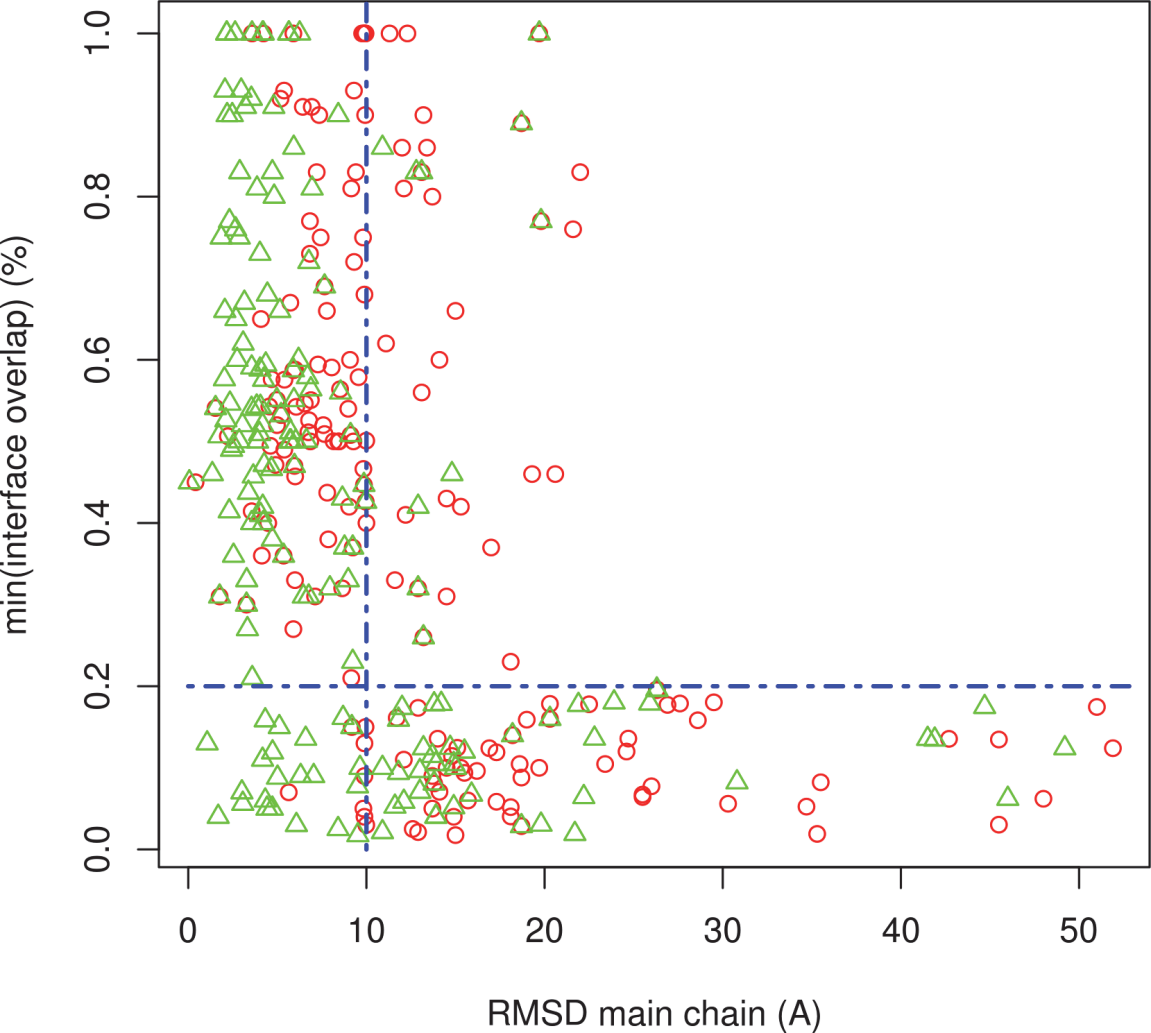
Relationship between l-RMSD (Ang) and interface coverage (%). RMSD was calculated using the main chain atoms. The interface coverage represents the lowest coverage of the predicted binding sites in either ligand / or receptor. Red empty circles and green empty triangles represent the best l-RMSD using all docking poses or the best poses among the top 200 clusters respectively.

Due to its nature, data-driven docking is less comprehensive than *ab initio* docking, i.e. data-driven docking directs the docking of receptor and ligand and thus restricts the search space. To further clarify the effect of the constraints imposed by the selection of interfaces and quality of the docking poses, we studied the relationship between the best l-RMSD and the overlap of the predicted and real interfaces (Fig. 2). As shown, only when the overlap of the predicted and native interface drops below 20% does the quality of docking models deteriorate substantially. Above 20% of interface overlap, V-D^2^OCK consistently samples docking poses below 10 Ang l-RMSD. These results agree with those previously reported by de Vries et al, which show that inclusion of a low proportion of native contacts is usually sufficient as the docking process can correct for the actual orientation of the proteins [8].

### Comparing VD^2^OCK and a competitive *ab initio* docking algorithm: ZDOCK

From the previous analysis, it can be concluded that the sampling of the docking space is efficient and generates docking poses close to the native ones, even though the search is directed by the predicted interfaces. The performance of the method was then assessed in terms of successful predictions among the top N, N being the number of predictions being considered. Three different scoring functions were considered: PatchDock native score [15], the ES3DC potential [27], and ZRANK score [28].

**Fig 3:**
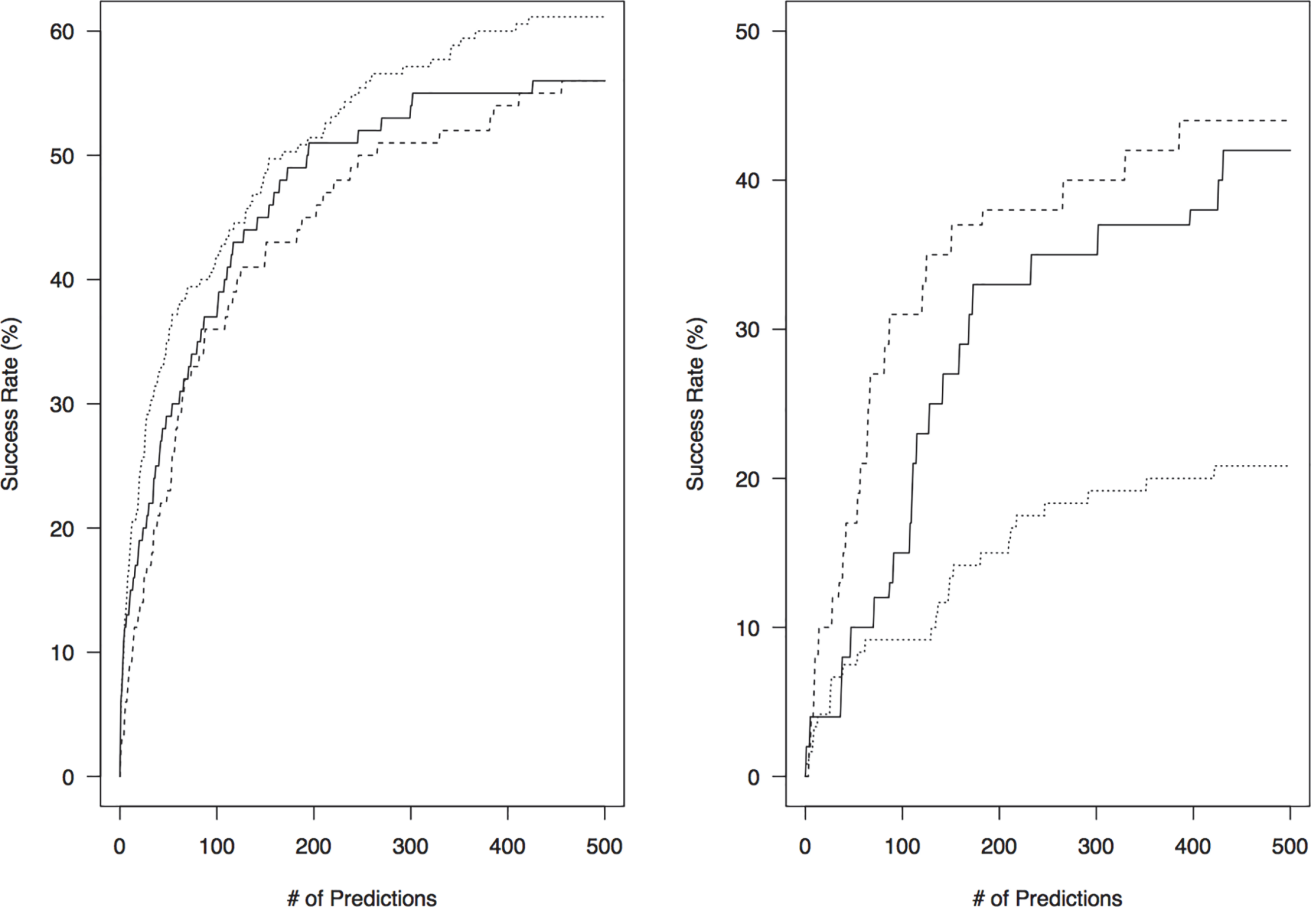
Success rates for all test cases (left) and medium/difficult cases (right) on Benchmark v4.0. PatchDock[15], ES3DC potential[27] and ZRANK scores[28] are shown as solid, dashed and dotted lines respectively.

The success rate is around 55% when considering the top 500 poses (Fig. 3) and 69% when considering only rigid-body, or easy, cases (S1 Fig.). The scoring function that performed the best was ZRANK, followed by PatchDock and ES3DC potential. However, ES3DC, a coarse-grained statistical potential, outperformed both ZRANK and PatchDock native scores for flexible/difficult cases with a success rate close to 45% in comparison to 19% for ZRANK (Fig. 3). In general, the performance achieved is similar, if not higher, than ZDOCK[13], an *ab initio* protein docking method that was also benchmarked using the same dataset: BO4 [20] (for exhaustive comparison of success rate curves see Fig. 1 in [13].)

### Examples of predicted complexes using VD^2^OCK

**Fig 4:**
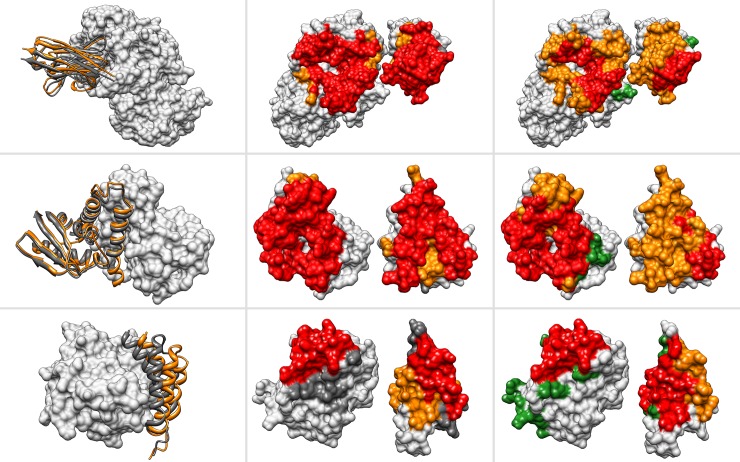
Examples of structural models. Rows (from top to bottom) show the comparison between native and predicted structures of protein complexes: camelid VHH domain and porcine pancreatic alpha-amylase (PDB code 1kxq)[29], BET3 and TPC6B core of TRAPP (PDB code 2cfh)[30], and Pol II epsilon and Hot proofreading complex (PDB code 2ido)[31]. Colums (from left to right) show: 1) the structure of native and predicted complex where receptor is depicted in surface (grey) and receptor as ribbon representation (native: dark grey; predicted: orange). 2) Surface representation of both receptor (left) and ligand (right) and the overlap (red) between native (dark grey) and predicted (orange). 3) Surface representation as in 2) showing the overlap (red) between predicted interface (green) and docking interface (orange).

Fig. 4 illustrates three different examples of predicted complexes one for each of the classes defined in B04 [20], i.e. easy, medium and difficult. These classes are defined depending on the level of conformational change upon formation of the protein complex: easy class is similar to rigid body docking (i.e. no conformation change); medium and difficult class implies conformational changes in the monomers upon binding. The first example, member of the ‘easy’ class in B04, is the protein complex formed by a camelid VHH domain bound to the porcine pancreatic alpha-amylase[29]. The second example represents a case of medium difficulty as per B04 classification and corresponds to the protein complex formed by a human Bet3 and Tpc6B of the transport protein particle complex[30]. Finally, the third example, the epsilon subunit of *E*.*coli* polymerase III in complex with the Hot protein[31], corresponds to the ‘difficult’ class.

For all the cases described above VD^2^OCK derived docking poses that closely resembles the structure of the respective native complexes. The superimposition of the native and predicted complexes (first column) and the overlap between the native and predicted interfaces (second column) show that the predicted structures closely resembles de native complex. In addition, the third column shows that the overlap between the V-PATCH predicted residues and the predicted complex interfaces is not total, showing that the docking is in fact correcting the final interface. These observations agree with previous[8] and our own observations in this work that shows that even with a low overlap between predicted and native interfaces the docking process can correct for the missing information(Fig. 2 and S1 Table).

### V-D^2^OCK web server

Although the computing time is not a measure of quality, it can limit the applicability of the method. In its current implementation, V-D^2^OCK requires 36 CPU hours on a standard desktop to complete the entire B04 dataset, i.e. 12 minutes per complex on average, including the prediction of interfaces, docking and clustering. Given the speed of the algorithm, V-D^2^OCK has been implemented as a web-application and predictions can be derived in real time (http://www.bioinsilico.org/VD2OCK). The web server provides a user-friendly interface to execute the docking algorithm and to analyse and visualize the structural models. As described, the number of potential docking poses can be large, even after applying the clustering step. The web application, however, features a bespoke viewer that allows easy navigation and visualization among the structural models. The structural models can be also sorted according to different criteria, which include the PatchDock (default), ZRANK and ES3DC scores, contact surface area, and cluster size. Finally, the coordinates of the docking poses, both centroids and poses within clusters, can be also downloaded.

## Conclusions

Here we describe V-D^2^OCK, a data-driven docking strategy that integrates V-PATCH, PatchDock[15] and a final clustering step. As shown, the method is able to sample suitable docking conformations even with low coverage of the native interfaces. The clustering step greatly reduces the number of docking poses with a limited impact on the quality of the models, facilitating the analysis and visualization of the docking solutions. We have explored different scoring functions and depending on the nature of the conformational change upon formation of the protein complex, ES3DC coarse-grained statistical potential performs better that ZRANK energy-based function. Finally, V-D^2^OCK is accessible via a web application, which features a bespoke molecular visualizer that allows users to easily and conveniently analyse, visualize and download the structural models of protein complexes. Moreover, users can select additional scoring functions and/or download the models generated by V-D^2^OCK and apply the scoring function of choice.

## Supporting Information

S1 Fig Success rates for easy cases (rigid-body) on Benchmark v.4.0. PatchDock[15], ES3DC potential[27] and ZRANK[28] scores are shown as solid, dashed and dotted lines respectively.
(TIFF)

S1 File Pseudo-code implementation of VPATCH algorithm. (DOCX)

S2 File URLs to download docking decoys derived for B04 using VD^2^OCK. (DOCX)

S1 Table VD^2^OCK predictions for protein complex on Benchmark v4.0. Columns represent the PDB code (first column), overlap between predicted and native interface in receptor (%; second column), overlap between predicted and native interface in ligand (%; third column), and l-RMSD (Ang) of the best docking pose (fourth column). The rest of the columns are grouped in 5 blocks of 3, each showing the l-RMSD (Ang) for the top scoring pose using PatchDock (PD)[15], ES3DC[27] and ZRANK (ZR)[28] scores within the TOP 1, TOP 10, TOP 50, TOP 100 and TOP 200 respectively. Blue, yellow and green blocks of the table show the easy, medium and difficult cases according to Benchmark v4.0 classification[20].
(DOCX)
