# Supplementary figures and images for: VORFFIP-Driven Dock: V-D2OCK, a Fast and Accurate Protein Docking Strategy
Source: PLoS One. 2015 Mar 12;10(3):e0118107. doi: 10.1371/journal.pone.0118107 (PMC4357426; doi:10.1371/journal.pone.0118107)

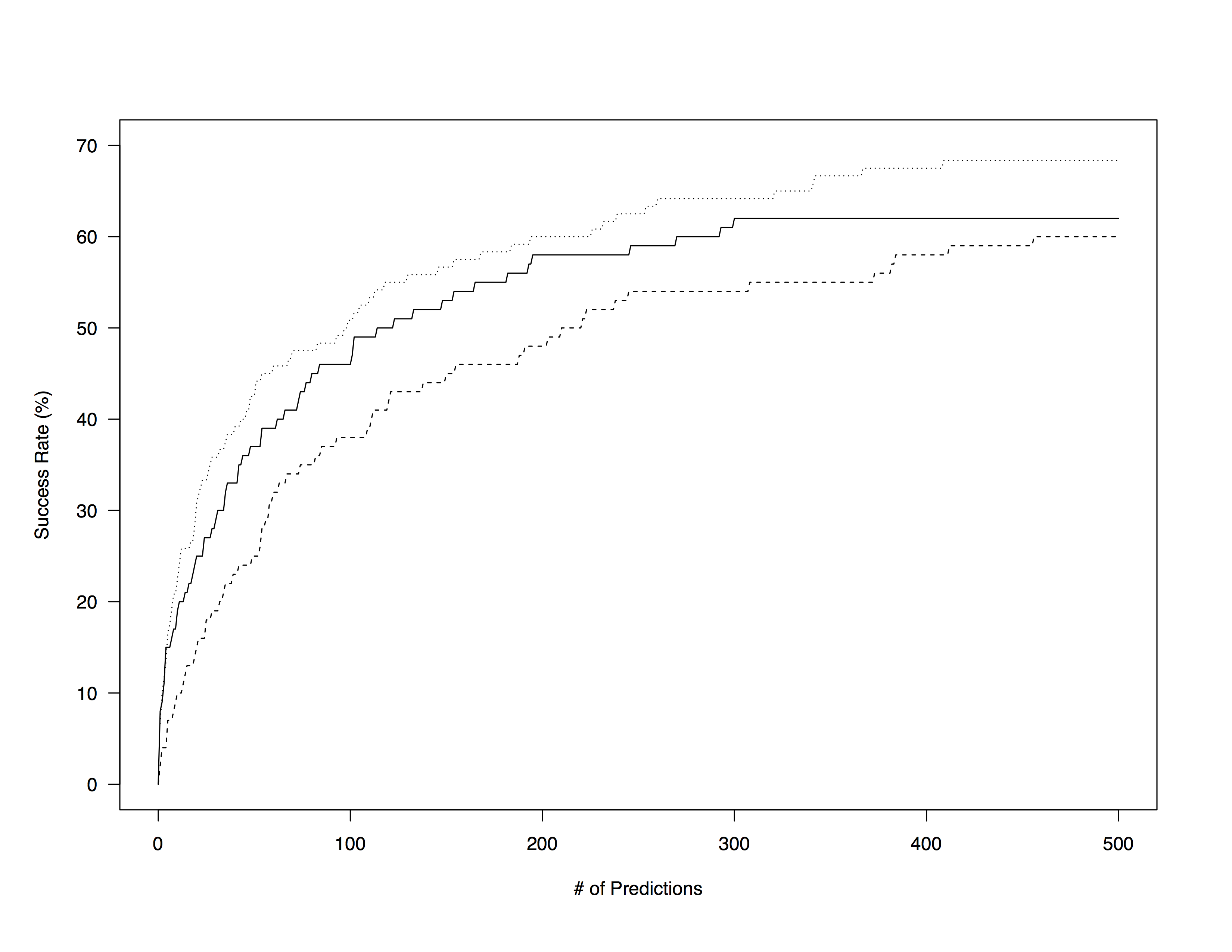

Supplement: S1 Fig — PatchDock[15], ES3DC potential[27] and ZRANK[28] scores are shown as solid, dashed and dotted lines respectively. (TIFF) [file pone.0118107.s001.tiff]
