# Supplementary material for: VORFFIP-Driven Dock: V-D2OCK, a Fast and Accurate Protein Docking Strategy
Source: PLoS One. 2015 Mar 12;10(3):e0118107. doi: 10.1371/journal.pone.0118107 (PMC4357426; doi:10.1371/journal.pone.0118107)
Supplement: S1 File — (DOCX) [file pone.0118107.s002.docx]

Supplementary Material on “**VORFFIP-driven docking: V-D^2^OCK, a fast and accurate protein docking strategy**” by Segura et al.

**MATERIAL AND METHODS**

**VPATCH algorithm**

The algorithm computes the interface patch by using VORFFIP scores. In the first step a new score, named *extended score*, is calculated for each residue. The extended score is a contribution of the predicted score for a particular residue and the environment score for the same residue. Let $\left\{ \left( a_{i},s_{i} \right);i=1,\ldots,N \right\}$ be the residues and predicted scores of a given protein, the extended score $s_{i}^{*}$ for a residue $a_{i}$ with neighbours $\left\{ a_{j};j=1,\ldots n \right\}$ is defined as

$$s_{i}^{*}=0.5[s_{i}^{'}+\sum_{j=1}^{n} c_{ij}s_{j}^{'}]$$

where$c_{ij}$ is the contact strength between $a_{i}$ and $a_{j}$ and $s_{i}^{'}$ is the normalized score calculated as

$$s_{i}^{'}=\frac{s_{i}-m}{M-m}$$

with$m=\min\left\{ s_{i};i=1,\ldots N \right\}$ and $m=\max\left\{ s_{i};i=1,\ldots N \right\}$.

The concept behind this approach is to start with the highest ranked residue, generate an initial patch and extend it to neighbouring residues until the score falls below a threshold. The process is divided in three steps: (i) Patch generation; (ii) Patch selection; and (iii) Patch extension.

*Patch generation*

Upon selecting the highest-ranking residue, the initial patch is generated by recursive selection of neighbouring residues that have a score above a certain threshold$\alpha$. The next pseudo-code algorithm computes this operation.

| 1: $\mathcal{N\leftarrow}$ neighbouring residues of $a_{i}$  2: **for each** non-marked $a_{j}$ in $\mathcal{N}$  3: **if** $s_{j}^{*}>\alpha$**then**  4: **for each** $a_{k}$ neighbour of $a_{j}$  5: **if** $a_{k}\mathcal{\notin N}$**then** add $a_{k}$ to $\mathcal{N}$  6: **mark**$a_{j}$  7: $\mathcal{P}_{i}\leftarrow$**set** $\mathcal{N}$ as the patch associated to $a_{i}$ |
| --- |

The parameter $\alpha$ is named the *hard average* cut-off and was calculated using the average of the extended scores for interface residues in the complexes of SOB4 dataset (see dataset section in the manuscript).

*Patch selection*

In this stage, patches of residues scored above the *hard average* $\alpha$ are removed and the redundancy generated by residues belonging to the same patch is simplified. The approach starts with a list of patches sorted by the patch size, then the patches associated to low scored residues are removed and for an accepted patch all other patches associated to its residues are excluded. The method can be implemented with the next pseudo-code algorithm.

| 1: $\mathcal{L \leftarrow}\left\{ \left( a_{i},\mathcal{P}_{i} \right) \right\}$ sort by $\left\vert\mathcal{P}_{i} \right\vert$  2: **for each** $(a_{i},\mathcal{P}_{i})$ in $\mathcal{L}$  3: **if** $s_{i}^{*}<\alpha$**then** remove $(a_{i},\mathcal{P}_{i})$ from $\mathcal{L}$  4: **else**  5: **for each** $a_{j}$ in $\mathcal{P}_{i}$  6: remove $(a_{j},\mathcal{P}_{j})$ from $\mathcal{L}$ |
| --- |

When the algorithm ends,$\mathcal{L}$ contains the list of selected patches that will be processed in the next step. The parameter $\alpha$ is the hard average used in the patch generation step of the algorithm.

*Patch extension*

The last stage of the algorithm extends the patches to maximize the size of the interface patch by including neighbouring residues that were not selected in the previous round and whose extended score is above a certain threshold $\beta$. The process was implemented with the next pseudo-code algorithm.

| 1: $\mathcal{G}_{i}\leftarrow\mathcal{P}_{i}$  2: **for each** $a_{j}$ in $\mathcal{P}_{i}$  3: **for each** $a_{k}$ neighbour of $a_{j}$  4: **if** $a_{k}\notin\mathcal{G}_{i}$ and $s_{k}^{*}>\beta$**then** add $a_{k}$ to $\mathcal{G}_{i}$  5: **mark** $\mathcal{G}_{i}$ as the extended patch |
| --- |

The parameter $\beta$ is named the *soft average* cut-off and was calculated by computing the average of extended scores in the case of residues that are not part of protein interfaces in SOB4 datase. The extended patches will form the list of residues that will be used to inform the docking (see main text).
