# Supplementary material for: VORFFIP-Driven Dock: V-D2OCK, a Fast and Accurate Protein Docking Strategy
Source: PLoS One. 2015 Mar 12;10(3):e0118107. doi: 10.1371/journal.pone.0118107 (PMC4357426; doi:10.1371/journal.pone.0118107)
Supplement: S2 File — (DOCX) [file pone.0118107.s003.docx]

Supplementary Material on “**VORFFIP-driven docking: V-D^2^OCK, a fast and accurate protein docking algorithm**” by Segura et al.

To download the entire set of docking poses and raw data please use the following link:

<http://www.bioinsilico.org/VD2OCK/PD_B4_results.tar.bz2>

PD_B4_results.tar.bz2: tar file compressed using bzip2

or the Harvard Dataverse Network:

<https://thedata.harvard.edu/dvn/dv/VD2OCK-B04> doi:10.7910/DVN/28610
