# Supplementary material for: VORFFIP-Driven Dock: V-D2OCK, a Fast and Accurate Protein Docking Strategy
Source: PLoS One. 2015 Mar 12;10(3):e0118107. doi: 10.1371/journal.pone.0118107 (PMC4357426; doi:10.1371/journal.pone.0118107)
Supplement: S1 Table — Columns represent the PDB code (first column), overlap between predicted and native interface in receptor (%; second column), overlap between predicted and native interface in ligand (%; third column), and l-RMSD (Ang) of the best docking pose (fourth column). The rest of the columns are grouped in 5 blocks of 3, each showing the l-RMSD (Ang) for the top scoring pose using PatchDock (PD)[15], ES3DC[27] and ZRANK (ZR)[28] scores within the TOP 1, TOP 10, TOP 50, TOP 100 and TOP 200 respectively. Blue, yellow and green blocks of the table show the easy, medium and difficult cases according to Benchmark v4.0 classification[20]. (DOCX) [file pone.0118107.s004.docx]

Supplementary Material on “**VORFFIP-driven docking: V-D^2^OCK, a fast and accurate protein docking strategy**” by Segura et al.

|  | BEST | REC | LIG |  | TOP 1 | | |  | TOP 10 | | |  | TOP 50 | | |  | TOP 100 | | |  | TOP 200 | | |
| --- | --- | --- | --- | --- | --- | --- | --- | --- | --- | --- | --- | --- | --- | --- | --- | --- | --- | --- | --- | --- | --- | --- | --- |
|  | RMSD | COV% | COV% |  | PD | S3DC | ZR |  | PD | S3DC | ZR |  | PD | S3DC | ZR |  | PD | S3DC | ZR |  | PD | S3DC | ZR |
| 1AHW | 22.2 | 61 | 4 |  | 50.7 | 37.7 | 57.0 |  | 40.7 | 31.1 | 39.4 |  | 27.8 | 26.2 | 30.1 |  | 27.8 | 22.7 | 30.1 |  | 26.8 | 22.7 | 27.8 |
| 1BVK | 3.21 | 71 | 90 |  | 46.3 | 30.0 | 24.7 |  | 22.4 | 28.7 | 24.7 |  | 12.5 | 24.0 | 12.9 |  | 8.67 | 14.5 | 8.76 |  | 8.67 | 14.2 | 8.76 |
| 1DQJ | 7.58 | 100 | 5 |  | 47.8 | 30.7 | 35.6 |  | 21.6 | 21.0 | 27.1 |  | 14.0 | 16.9 | 13.7 |  | 14.0 | 15.7 | 13.7 |  | 14.0 | 7.58 | 13.7 |
| 1E6J | 4.80 | 100 | 36 |  | 47.1 | 41.0 | 26.3 |  | 21.7 | 15.4 | 5.35 |  | 13.0 | 13.8 | 5.35 |  | 5.35 | 9.4 | 5.35 |  | 5.35 | 8.37 | 5.35 |
| 1JPS | 6.78 | 33 | 44 |  | 38.3 | 63.5 | 47.9 |  | 21.8 | 25.4 | 21.4 |  | 8.20 | 23.4 | 21.4 |  | 6.78 | 16.3 | 21.4 |  | 6.78 | 16.3 | 11.2 |
| 1MLC | 3.86 | 92 | 81 |  | 51.7 | 28.1 | 18.3 |  | 16.4 | 20.0 | 3.86 |  | 3.86 | 6.59 | 3.86 |  | 3.86 | 6.59 | 3.86 |  | 3.86 | 6.59 | 3.86 |
| 1VFB | 4.23 | 100 | 91 |  | 46.8 | 34.4 | 33.8 |  | 24.3 | 23.4 | 33.5 |  | 8.29 | 20.5 | 14.0 |  | 8.29 | 20.5 | 5.58 |  | 8.29 | 17.8 | 5.58 |
| 1WEJ | 11.9 | 90 | 7 |  | 29.7 | 15.4 | 35.4 |  | 15.0 | 15.4 | 19.4 |  | 15.0 | 15.4 | 18.7 |  | 15.0 | 15.0 | 17.7 |  | 15.0 | 11.9 | 14.1 |
| 2FD6 | 18.1 | 92 | 23 |  | 27.8 | 42.8 | 30.7 |  | 18.1 | 29.8 | 28.5 |  | 18.1 | 18.1 | 25.7 |  | 18.1 | 18.1 | 23.7 |  | 18.1 | 18.1 | 18.1 |
| 2I25 | 3.59 | 46 | 33 |  | 28.7 | 13.4 | 20.6 |  | 20.4 | 13.4 | 20.6 |  | 20.4 | 12.3 | 19.1 |  | 5.12 | 12.3 | 17.3 |  | 5.12 | 11.5 | 5.12 |
| 2VIS | 58.1 | 90 | 1 |  | 87.0 | 76.7 | 79.7 |  | 58.1 | 59.3 | 59.3 |  | 58.1 | 58.1 | 58.1 |  | 58.1 | 58.1 | 58.1 |  | 58.1 | 58.1 | 58.1 |
| 1BJ1 | 5.01 | 93 | 94 |  | 60.3 | 60.8 | 48.9 |  | 40.9 | 33.7 | 38.1 |  | 24.9 | 24.4 | 24.8 |  | 20.2 | 5.01 | 24.8 |  | 18.7 | 5.01 | 17.1 |
| 1FSK | 2.16 | 100 | 100 |  | 56.3 | 27.5 | 30.8 |  | 18.2 | 24.3 | 21.8 |  | 5.24 | 19.5 | 6.69 |  | 5.24 | 17.9 | 6.69 |  | 5.24 | 13.8 | 6.69 |
| 1I9R | 9.11 | 43 | 30 |  | 92.4 | 43.1 | 100 |  | 53.2 | 23.4 | 90.0 |  | 9.11 | 18.2 | 36.0 |  | 9.11 | 9.11 | 23.8 |  | 9.11 | 9.11 | 18.2 |
| 1IQD | 5.90 | 33 | 86 |  | 48.2 | 36.3 | 31.5 |  | 44.0 | 22.0 | 22.7 |  | 18.7 | 17.2 | 9.65 |  | 16.8 | 17.2 | 9.65 |  | 5.90 | 5.90 | 9.65 |
| 1K4C | 13.8 | 68 | 8 |  | 23.3 | 51.5 | 32.7 |  | 23.3 | 34.8 | 32.7 |  | 23.3 | 21.3 | 26.7 |  | 23.3 | 19.3 | 23.8 |  | 18.5 | 19.3 | 19.3 |
| 1KXQ | 4.17 | 61 | 52 |  | 26.2 | 37.8 | 4.98 |  | 4.98 | 6.03 | 4.98 |  | 4.98 | 6.03 | 4.98 |  | 4.98 | 6.03 | 4.98 |  | 4.98 | 6.03 | 4.98 |
| 1NCA | 14.9 | 83 | 9 |  | 30.7 | 36.7 | 40.8 |  | 25.5 | 15.1 | 29.2 |  | 19.2 | 15.1 | 14.9 |  | 14.9 | 15.1 | 14.9 |  | 14.9 | 14.9 | 14.9 |
| 1NSN | 15.1 | 41 | 3 |  | 18.5 | 40.5 | 23.3 |  | 16.2 | 19.2 | 22.8 |  | 15.6 | 19.2 | 17.7 |  | 15.6 | 15.1 | 17.7 |  | 15.1 | 15.1 | 16.2 |
| 1QFW | 20.6 | 100 | 46 |  | 53.9 | 45.9 | 63.5 |  | 34.6 | 39.4 | 43.4 |  | 31.8 | 39.4 | 29.7 |  | 25.6 | 34.4 | 29.7 |  | 25.6 | 24.3 | 29.7 |
| 2QFW | 4.21 | 100 | 100 |  | 72.6 | 66.8 | 38.8 |  | 31.7 | 43.5 | 18.2 |  | 28.2 | 27.1 | 5.50 |  | 25.3 | 21.2 | 5.50 |  | 5.90 | 12.1 | 5.50 |
| 2JEL | 12.7 | 100 | 6 |  | 52.3 | 17.7 | 16.8 |  | 16.5 | 17.7 | 16.8 |  | 15.5 | 17.7 | 16.0 |  | 15.1 | 16.5 | 14.8 |  | 14.2 | 14.8 | 14.8 |
| 1AVX | 2.06 | 93 | 100 |  | 25.2 | 46.0 | 7.77 |  | 12.3 | 24.5 | 7.77 |  | 12.3 | 11.8 | 7.77 |  | 11.3 | 9.31 | 7.77 |  | 11.3 | 9.31 | 7.77 |
| 1AY7 | 3.27 | 60 | 100 |  | 21.2 | 28.2 | 39.6 |  | 10 | 25.1 | 19.9 |  | 10 | 16.4 | 3.27 |  | 3.27 | 9.43 | 3.27 |  | 3.27 | 9.3 | 3.27 |
| 1BVN | 2.86 | 75 | 100 |  | 9.8 | 36.2 | 20.1 |  | 9.8 | 27.2 | 10.0 |  | 9.6 | 21.0 | 7.17 |  | 9.6 | 15.1 | 7.17 |  | 9.1 | 6.07 | 7.17 |
| 1CGI | 3.55 | 54 | 100 |  | 17.5 | 15.6 | 5.23 |  | 14.8 | 5.23 | 5.23 |  | 8.98 | 5.23 | 5.23 |  | 8.98 | 5.23 | 5.23 |  | 8.98 | 5.23 | 5.23 |
| 1CLV | 2.74 | 60 | 100 |  | 9.74 | 24.4 | 13.5 |  | 9.6 | 8.99 | 12.0 |  | 9.2 | 8.04 | 9.7 |  | 9.2 | 8.04 | 9.2 |  | 9.2 | 8.04 | 9.2 |
| 1D6R | 4.82 | 100 | 100 |  | 15.6 | 25.8 | 38.7 |  | 15.6 | 23.4 | 18.4 |  | 14.3 | 17.2 | 14.2 |  | 11.8 | 6.98 | 11.9 |  | 6.98 | 6.98 | 9.21 |
| 1DFJ | 33.8 | 3 | 61 |  | 42.1 | 51.2 | 73.3 |  | 35.5 | 50.1 | 50.0 |  | 35.5 | 50.1 | 39.5 |  | 35.5 | 50.1 | 38.6 |  | 35.5 | 38.6 | 38.6 |
| 1E6E | 4.55 | 42 | 64 |  | 33.4 | 30.1 | 55.0 |  | 8.49 | 5.92 | 8.94 |  | 7.92 | 5.92 | 7.28 |  | 7.92 | 5.92 | 7.28 |  | 7.92 | 5.92 | 7.28 |
| 1EAW | 2.89 | 100 | 83 |  | 16.8 | 28.5 | 33.7 |  | 16.1 | 15.9 | 8.74 |  | 4.99 | 12.7 | 8.74 |  | 4.99 | 9.21 | 8.74 |  | 4.99 | 9.21 | 8.74 |
| 1EWY | 3.56 | 63 | 40 |  | 18.7 | 31.9 | 13.9 |  | 6.61 | 18.4 | 8.39 |  | 6.61 | 6.61 | 8.12 |  | 6.61 | 6.61 | 7.59 |  | 6.61 | 6.61 | 7.59 |
| 1EZU | 7.65 | 69 | 95 |  | 15.5 | 78.2 | 40.7 |  | 15.5 | 37.9 | 37.8 |  | 10.0 | 9.9 | 37.1 |  | 10.0 | 9.9 | 21.8 |  | 7.65 | 9.9 | 14.6 |
| 1F34 | 2.54 | 36 | 65 |  | 33.9 | 35.8 | 33.6 |  | 20.0 | 19.1 | 29.9 |  | 4.13 | 8.18 | 3.45 |  | 4.13 | 8.18 | 3.45 |  | 4.13 | 8.18 | 3.45 |
| 1FLE | 2.47 | 90 | 100 |  | 17.4 | 23.5 | 8.11 |  | 15.4 | 22.6 | 8.11 |  | 8.80 | 16.4 | 8.11 |  | 8.80 | 6.60 | 8.11 |  | 8.26 | 6.60 | 8.11 |
| 1GL1 | 2.05 | 100 | 66 |  | 39.3 | 21.1 | 21.5 |  | 5.44 | 13.0 | 9.1 |  | 5.44 | 6.33 | 9.1 |  | 5.44 | 6.33 | 9.1 |  | 5.44 | 6.33 | 9.1 |
| 1GXD | 6.59 | 66 | 95 |  | 80.3 | 41.3 | 18.3 |  | 66.0 | 20.1 | 16.8 |  | 36.1 | 19.0 | 9.19 |  | 36.1 | 17.1 | 9.19 |  | 30.3 | 13.9 | 9.19 |
| 1HIA | 7.95 | 32 | 100 |  | 8.64 | 37.2 | 34.4 |  | 8.64 | 19.9 | 15.7 |  | 8.64 | 19.0 | 9.4 |  | 8.64 | 11.3 | 9.4 |  | 8.64 | 8.96 | 9.4 |
| 1JTG | 2.68 | 65 | 100 |  | 4.08 | 32.7 | 50.7 |  | 4.08 | 25.7 | 18.8 |  | 4.08 | 16.9 | 4.08 |  | 4.08 | 9.1 | 4.08 |  | 4.08 | 9.1 | 4.08 |
| 1MAH | 1.85 | 75 | 100 |  | 47.8 | 60.9 | 52.8 |  | 23.9 | 20.1 | 7.43 |  | 7.43 | 14.0 | 6.58 |  | 7.43 | 8.89 | 6.58 |  | 7.43 | 1.85 | 6.58 |
| 1N8O | 3.58 | 100 | 100 |  | 28.9 | 50.9 | 44.4 |  | 18.0 | 17.5 | 9.42 |  | 14.2 | 10.0 | 9.42 |  | 3.58 | 10.0 | 9.42 |  | 3.58 | 10.0 | 9.42 |
| 1OC0 | 3.73 | 21 | 80 |  | 39.0 | 56.7 | 17.7 |  | 17.3 | 37.6 | 7.25 |  | 8.31 | 9.2 | 3.73 |  | 8.31 | 9.2 | 3.73 |  | 7.25 | 7.25 | 3.73 |
| 1OPH | 9.9 | 100 | 86 |  | 40.9 | 29.1 | 42.9 |  | 23.3 | 17.5 | 29.3 |  | 22.5 | 15.2 | 9.9 |  | 12.0 | 9.9 | 9.9 |  | 12.0 | 9.9 | 9.9 |
| 1OYV | 3.15 | 67 | 94 |  | 19.8 | 20.5 | 32.4 |  | 17.1 | 15.8 | 7.17 |  | 8.65 | 12.6 | 7.17 |  | 8.65 | 12.6 | 7.17 |  | 8.65 | 12.6 | 7.17 |
| BOYV | 13.0 | 80 | 100 |  | 31.8 | 49.0 | 27.0 |  | 18.8 | 23.5 | 27.0 |  | 16.5 | 17.2 | 20.1 |  | 16.5 | 15.0 | 19.4 |  | 15.5 | 15.0 | 14.7 |
| 1PPE | 4.17 | 100 | 100 |  | 9.57 | 21.7 | 4.17 |  | 4.17 | 7.42 | 4.17 |  | 4.17 | 7.42 | 4.17 |  | 4.17 | 7.42 | 4.17 |  | 4.17 | 7.42 | 4.17 |
| 1R0R | 2.16 | 100 | 100 |  | 15.3 | 24.5 | 16.1 |  | 9.3 | 13.1 | 14.0 |  | 7.74 | 5.70 | 6.50 |  | 7.74 | 5.70 | 6.50 |  | 7.74 | 5.70 | 6.50 |
| 1TMQ | 2.03 | 54 | 90 |  | 2.03 | 27.7 | 19.4 |  | 2.03 | 13.4 | 12.6 |  | 2.03 | 5.78 | 4.41 |  | 2.03 | 5.78 | 4.41 |  | 2.03 | 5.78 | 4.41 |
| 1UDI | 2.31 | 95 | 77 |  | 30.4 | 29.8 | 27.2 |  | 21.9 | 13.4 | 19.7 |  | 6.83 | 7.18 | 19.7 |  | 6.83 | 7.18 | 6.55 |  | 6.83 | 2.31 | 6.55 |
| 1YVB | 0.07 | 45 | 100 |  | 2.90 | 2.18 | 8.19 |  | 0.99 | 2.18 | 0.69 |  | 0.95 | 1.36 | 0.07 |  | 0.94 | 1.35 | 0.07 |  | 0.41 | 0.78 | 0.07 |
| 2ABZ | 4.60 | 91 | 100 |  | 22.1 | 33.8 | 34.4 |  | 18.0 | 19.2 | 16.2 |  | 4.60 | 15.4 | 14.8 |  | 4.60 | 15.4 | 14.8 |  | 4.60 | 9.7 | 12.6 |
| 2J0T | 5.70 | 50 | 100 |  | 21.0 | 24.4 | 43.1 |  | 11.3 | 15.8 | 23.6 |  | 11.3 | 15.5 | 9.21 |  | 5.70 | 12.0 | 5.70 |  | 5.70 | 5.70 | 5.70 |
| 2MTA | 2.54 | 21 | 84 |  | 55.4 | 51.9 | 21.4 |  | 8.63 | 50.2 | 7.76 |  | 8.63 | 13.5 | 7.76 |  | 8.56 | 5.70 | 4.94 |  | 7.66 | 3.69 | 4.94 |
| 2O8V | 8.69 | 100 | 87 |  | 43.3 | 24.4 | 20.4 |  | 32.0 | 23.5 | 20.4 |  | 12.8 | 12.5 | 11.2 |  | 12.8 | 12.4 | 11.2 |  | 12.8 | 12.4 | 11.2 |
| 2OUL | 3.18 | 52 | 91 |  | 24.2 | 17.9 | 56.1 |  | 15.1 | 13.8 | 3.99 |  | 6.86 | 4.8 | 3.99 |  | 6.86 | 4.8 | 3.99 |  | 3.99 | 4.8 | 3.99 |
| 2PCC | 6.03 | 50 | 100 |  | 40.0 | 12.1 | 38.9 |  | 18.0 | 8.95 | 18.4 |  | 12.1 | 8.41 | 11.5 |  | 8.41 | 8.41 | 11.5 |  | 8.41 | 8.41 | 8.41 |
| 2SIC | 3.21 | 91 | 100 |  | 20.8 | 21.8 | 6.43 |  | 17.7 | 18.2 | 6.43 |  | 6.43 | 9.71 | 6.43 |  | 6.43 | 9.71 | 3.21 |  | 6.43 | 9.6 | 3.21 |
| 2SNI | 4.67 | 71 | 100 |  | 9.5 | 37.1 | 19.0 |  | 9.5 | 15.5 | 4.67 |  | 9.5 | 4.67 | 4.67 |  | 9.3 | 4.67 | 4.67 |  | 9.3 | 4.67 | 4.67 |
| 2UUY | 5.92 | 86 | 100 |  | 19.6 | 30.2 | 28.8 |  | 17.9 | 17.1 | 15.8 |  | 13.4 | 14.3 | 13.5 |  | 13.4 | 14.3 | 13.5 |  | 5.92 | 11.2 | 5.92 |
| 3SGQ | 3.04 | 52 | 91 |  | 17.7 | 25.6 | 5.99 |  | 8.07 | 9.2 | 5.99 |  | 8.07 | 9.2 | 3.04 |  | 8.07 | 9.2 | 3.04 |  | 8.07 | 8.73 | 3.04 |
| 7CEI | 4.24 | 100 | 9 |  | 38.8 | 32.3 | 26.5 |  | 25.9 | 15.8 | 16.1 |  | 9.4 | 15.8 | 8.65 |  | 9.4 | 8.65 | 8.65 |  | 9.4 | 8.65 | 8.65 |
| 1A2K | 4.82 | 80 | 100 |  | 59.0 | 52.7 | 24.6 |  | 15.1 | 19.7 | 22.2 |  | 15.1 | 4.82 | 11.9 |  | 13.7 | 4.82 | 11.9 |  | 9.7 | 4.82 | 4.82 |
| 1AK4 | 14.8 | 42 | 100 |  | 30.1 | 26.8 | 40.8 |  | 27.6 | 17.3 | 21.0 |  | 15.3 | 15.3 | 19.5 |  | 15.3 | 14.8 | 14.8 |  | 14.8 | 14.8 | 14.8 |
| 1AKJ | 26.3 | 5 | 30 |  | 63.8 | 57.4 | 69.6 |  | 41.5 | 53.6 | 58.0 |  | 41.5 | 48.5 | 48.5 |  | 41.5 | 48.5 | 36.9 |  | 41.5 | 48.5 | 36.9 |
| 1AZS | 9.2 | 100 | 100 |  | 71.2 | 74.9 | 67.1 |  | 42.1 | 33.3 | 9.2 |  | 40.5 | 30.2 | 9.2 |  | 20.6 | 20.6 | 9.2 |  | 9.2 | 9.2 | 9.2 |
| 1B6C | 2.97 | 100 | 93 |  | 34.5 | 45.7 | 35.3 |  | 5.38 | 23.0 | 12.4 |  | 5.38 | 6.99 | 5.38 |  | 5.38 | 6.99 | 5.38 |  | 5.38 | 6.99 | 5.38 |
| 1BUH | 3.72 | 100 | 100 |  | 36.0 | 43.7 | 24.8 |  | 33.2 | 19.6 | 13.6 |  | 18.8 | 15.4 | 7.24 |  | 17.0 | 11.2 | 7.24 |  | 7.64 | 11.2 | 7.24 |
| 1E96 | 2.43 | 41 | 63 |  | 36.2 | 42.6 | 37.7 |  | 31.3 | 32.1 | 6.34 |  | 29.1 | 18.5 | 5.22 |  | 13.7 | 18.5 | 5.22 |  | 9.9 | 13.7 | 5.22 |
| 1EFN | 6.95 | 81 | 100 |  | 40.5 | 22.6 | 31.1 |  | 27.3 | 12.0 | 24.1 |  | 15.4 | 11.3 | 15.3 |  | 13.2 | 11.3 | 9.5 |  | 7.56 | 9.6 | 9.5 |
| 1F51 | 3.01 | 57 | 66 |  | 19.6 | 39.6 | 51.7 |  | 16.5 | 20.7 | 46.7 |  | 10 | 11.6 | 12.3 |  | 6.87 | 8.89 | 12.3 |  | 5.64 | 8.89 | 4.34 |
| 1FC2 | 12.9 | 81 | 100 |  | 55.4 | 51.7 | 55.4 |  | 43.3 | 42.8 | 37.4 |  | 26.5 | 25.8 | 13.6 |  | 15.2 | 13.6 | 13.6 |  | 12.9 | 13.6 | 12.9 |
| 1FCC | 5.21 | 91 | 62 |  | 43.5 | 72.0 | 45.2 |  | 39.0 | 21.1 | 37.4 |  | 28.1 | 9.2 | 25.9 |  | 17.3 | 9.2 | 18.0 |  | 16.8 | 9.2 | 12.8 |
| 1FFW | 3.57 | 83 | 100 |  | 18.7 | 41.3 | 23.3 |  | 14.7 | 37.2 | 17.2 |  | 5.34 | 23.4 | 11.1 |  | 5.34 | 18.0 | 9.4 |  | 5.34 | 9.4 | 8.62 |
| 1FQJ | 8.65 | 43 | 56 |  | 42.1 | 53.0 | 50.0 |  | 26.6 | 38.3 | 25.8 |  | 20.4 | 20.4 | 20.4 |  | 20.4 | 8.65 | 20.4 |  | 14.5 | 8.65 | 20.4 |
| 1GCQ | 2.73 | 100 | 72 |  | 22.5 | 58.0 | 37.6 |  | 19.6 | 54.0 | 36.6 |  | 18.4 | 17.9 | 2.73 |  | 18.4 | 16.4 | 2.73 |  | 13.2 | 16.2 | 2.73 |
| 1GHQ | 11.9 | 28 | 2 |  | 57.7 | 60.4 | 56.0 |  | 47.4 | 59.2 | 35.5 |  | 28.9 | 36.3 | 21.4 |  | 27.3 | 27.2 | 21.4 |  | 14.7 | 25.2 | 21.4 |
| 1GLA | 13.9 | 7 | 64 |  | 49.4 | 24.1 | 54.8 |  | 34.9 | 24.1 | 32.2 |  | 25.0 | 17.5 | 19.2 |  | 24.8 | 16.4 | 16.6 |  | 18.8 | 16.4 | 16.6 |
| 1GPW | 22.3 | 8 | 68 |  | 57.3 | 31.4 | 54.7 |  | 26.2 | 25.5 | 40.7 |  | 26.2 | 25.5 | 32.7 |  | 26.2 | 25.5 | 27.7 |  | 26.2 | 22.9 | 25.5 |
| 1H9D | 4.72 | 100 | 38 |  | 28.5 | 9.8 | 19.2 |  | 27.8 | 9.8 | 13.4 |  | 26.6 | 9.8 | 13.4 |  | 7.86 | 9.8 | 9.27 |  | 7.86 | 7.61 | 9.27 |
| 1HCF | 6.69 | 61 | 100 |  | 50.1 | 62.8 | 51.6 |  | 23.5 | 22.4 | 23.8 |  | 12.0 | 9.9 | 17.3 |  | 9.56 | 9.9 | 9.2 |  | 7.33 | 9.9 | 9.2 |
| 1HE1 | 5.11 | 15 | 70 |  | 6.19 | 55.7 | 29.0 |  | 6.19 | 42.7 | 16.7 |  | 6.19 | 21.4 | 16.3 |  | 6.19 | 20.7 | 16.0 |  | 6.19 | 16.8 | 16.0 |
| 1I4D | 9.1 | 7 | 61 |  | 77.8 | 59.2 | 64.2 |  | 12.1 | 19.9 | 26.2 |  | 12.1 | 13.4 | 16.5 |  | 12.1 | 12.6 | 16.3 |  | 12.1 | 12.1 | 16.3 |
| 1J2J | 2.63 | 100 | 100 |  | 31.5 | 44.9 | 38.4 |  | 20.3 | 9.33 | 8.28 |  | 8.11 | 6.59 | 6.63 |  | 8.11 | 6.59 | 6.63 |  | 8.11 | 6.59 | 6.63 |
| 1JWH | 13.1 | 100 | 83 |  | 57.9 | 95.4 | 42.0 |  | 42.7 | 30.2 | 29.6 |  | 29.7 | 23.9 | 13.1 |  | 25.2 | 16.6 | 13.1 |  | 25.2 | 16.6 | 13.1 |
| 1K74 | 23.2 | 56 | 3 |  | 33.1 | 70.3 | 54.2 |  | 31.0 | 45.5 | 33.1 |  | 28.1 | 31.0 | 28.4 |  | 26.9 | 31.0 | 28.4 |  | 26.9 | 25.2 | 28.4 |
| 1KAC | 28.4 | 61 | 76 |  | 46.9 | 50.1 | 56.0 |  | 35.3 | 43.9 | 49.1 |  | 35.3 | 36.1 | 39.2 |  | 32.1 | 36.1 | 32.1 |  | 32.1 | 32.2 | 32.1 |
| 1KLU | 24.0 | 7 | 81 |  | 41.4 | 55.8 | 56.2 |  | 32.0 | 39.2 | 45.9 |  | 24.6 | 33.9 | 31.7 |  | 24.6 | 27.8 | 25.4 |  | 24.6 | 27.8 | 25.4 |
| 1KTZ | 4.73 | 100 | 100 |  | 41.2 | 44.7 | 33.2 |  | 36.3 | 44.7 | 27.4 |  | 30.9 | 38.8 | 21.1 |  | 20.2 | 37.3 | 11.3 |  | 18.6 | 37.3 | 11.3 |
| 1KXP | 9.23 | 52 | 54 |  | 80.6 | 79.5 | 92.2 |  | 27.8 | 44.0 | 66.3 |  | 9.23 | 9.23 | 27.8 |  | 9.23 | 9.23 | 27.8 |  | 9.23 | 9.23 | 9.23 |
| 1ML0 | 3.45 | 88 | 33 |  | 24.1 | 64.0 | 58.4 |  | 3.56 | 20.8 | 3.45 |  | 3.56 | 15.0 | 3.45 |  | 3.56 | 9.2 | 3.45 |  | 3.56 | 4.70 | 3.45 |
| 1OFU | 8.42 | 100 | 90 |  | 49.1 | 70.6 | 88.1 |  | 49.1 | 49.8 | 29.6 |  | 34.9 | 27.8 | 8.42 |  | 14.5 | 27.8 | 8.42 |  | 9.1 | 27.3 | 8.42 |
| 1PVH | 9.1 | 100 | 37 |  | 31.2 | 33.8 | 44.1 |  | 18.4 | 9.9 | 15.8 |  | 18.1 | 9.9 | 12.1 |  | 17.0 | 9.9 | 12.1 |  | 14.5 | 9.9 | 12.1 |
| 1QA9 | 44.6 | 7 | 6 |  | 84.5 | 100 | 98.7 |  | 55.6 | 75.0 | 65.4 |  | 47.8 | 56.6 | 59.4 |  | 47.8 | 55.4 | 50.1 |  | 47.8 | 48.4 | 50.1 |
| 1RLB | 4.97 | 38 | 100 |  | 66.8 | 76.8 | 84.1 |  | 45.5 | 20.5 | 9.95 |  | 26.7 | 20.5 | 9.95 |  | 21.8 | 20.5 | 9.95 |  | 17.1 | 4.97 | 4.97 |
| 1RV6 | 4.03 | 100 | 100 |  | 46.1 | 38.9 | 53.7 |  | 41.8 | 32.7 | 19.4 |  | 17.6 | 17.5 | 12.7 |  | 5.99 | 17.5 | 4.03 |  | 5.99 | 5.31 | 4.03 |
| 1S1Q | 2.96 | 63 | 27 |  | 22.9 | 15.3 | 25.6 |  | 9.8 | 15.3 | 18.3 |  | 5.90 | 9.9 | 12.4 |  | 5.90 | 9.9 | 8.31 |  | 5.90 | 2.96 | 8.31 |
| 1SBB | 16.6 | 2 | 83 |  | 56.2 | 41.9 | 73.7 |  | 55.0 | 35.1 | 50.9 |  | 45.1 | 21.2 | 40.7 |  | 29.4 | 16.6 | 31.0 |  | 25.8 | 16.6 | 24.4 |
| 1T6B | 7.06 | 94 | 41 |  | 47.5 | 37.2 | 35.1 |  | 20.3 | 26.5 | 21.8 |  | 13.8 | 20.1 | 17.6 |  | 13.8 | 11.9 | 16.0 |  | 8.05 | 8.45 | 7.06 |
| 1US7 | 17.3 | 25 | 10 |  | 32.3 | 37.0 | 39.2 |  | 24.7 | 26.1 | 34.9 |  | 20.2 | 23.0 | 22.6 |  | 20.2 | 20.4 | 21.8 |  | 20.2 | 17.3 | 18.8 |
| 1WDW | 1.76 | 31 | 51 |  | 20.6 | 9.05 | 20.6 |  | 1.76 | 8.80 | 19.4 |  | 1.76 | 8.80 | 2.05 |  | 1.76 | 8.80 | 1.76 |  | 1.76 | 8.80 | 1.76 |
| 1XD3 | 3.89 | 50 | 100 |  | 13.2 | 16.4 | 29.1 |  | 8.55 | 3.89 | 12.9 |  | 8.55 | 3.89 | 6.52 |  | 8.55 | 3.89 | 6.52 |  | 8.55 | 3.89 | 6.52 |
| 1XU1 | 2.09 | 22 | 92 |  | 44.4 | 22.1 | 31.8 |  | 13.2 | 22.1 | 24.9 |  | 10.0 | 19.9 | 18.5 |  | 10.0 | 15.9 | 13.2 |  | 9.1 | 14.3 | 5.80 |
| 1Z0K | 2.18 | 100 | 90 |  | 34.3 | 45.9 | 13.0 |  | 14.5 | 11.5 | 13.0 |  | 9.5 | 9.7 | 6.97 |  | 9.5 | 9.7 | 6.97 |  | 9.5 | 9.7 | 6.97 |
| 1Z5Y | 6.28 | 100 | 100 |  | 59.9 | 47.7 | 30.7 |  | 27.2 | 11.4 | 9.71 |  | 13.5 | 11.4 | 9.71 |  | 13.5 | 11.4 | 9.71 |  | 13.0 | 8.12 | 9.71 |
| 1ZHH | 15.0 | 8 | 36 |  | 66.5 | 51.6 | 51.4 |  | 25.6 | 51.6 | 31.9 |  | 25.6 | 34.9 | 29.3 |  | 25.6 | 31.4 | 29.3 |  | 17.3 | 28.4 | 26.1 |
| 1ZHI | 4.05 | 33 | 57 |  | 43.0 | 19.1 | 35.4 |  | 31.9 | 18.3 | 17.7 |  | 22.0 | 18.3 | 4.87 |  | 11.8 | 9.1 | 4.87 |  | 11.8 | 9.1 | 4.87 |
| 2A5T | 6.46 | 45 | 36 |  | 63.8 | 75.0 | 45.2 |  | 25.3 | 22.5 | 14.8 |  | 14.6 | 22.5 | 14.8 |  | 14.6 | 22.5 | 14.8 |  | 13.7 | 10.0 | 14.8 |
| 2A9K | 5.96 | 47 | 78 |  | 21.3 | 51.6 | 32.4 |  | 11.6 | 20.2 | 21.0 |  | 11.6 | 17.0 | 21.0 |  | 11.6 | 17.0 | 19.9 |  | 5.96 | 5.96 | 5.96 |
| 2AJF | 14.2 | 12 | 57 |  | 63.9 | 51.7 | 61.8 |  | 62.5 | 26.4 | 35.2 |  | 20.3 | 26.4 | 19.1 |  | 20.3 | 26.4 | 14.4 |  | 19.4 | 19.9 | 14.4 |
| 2B4J | 5.14 | 66 | 100 |  | 21.6 | 62.8 | 73.3 |  | 21.6 | 30.9 | 23.7 |  | 21.6 | 23.6 | 14.7 |  | 15.0 | 21.8 | 13.1 |  | 12.1 | 15.6 | 10 |
| 2BTF | 4.02 | 73 | 73 |  | 51.5 | 72.9 | 39.6 |  | 50.7 | 22.6 | 18.9 |  | 9.06 | 13.1 | 9.92 |  | 6.83 | 13.1 | 9.5 |  | 6.83 | 7.72 | 9.5 |
| 2FJU | 51.9 | 2 | 100 |  | 56.0 | 65.0 | 77.3 |  | 53.8 | 54.9 | 55.9 |  | 52.5 | 52.6 | 55.9 |  | 52.5 | 52.6 | 53.8 |  | 51.9 | 52.6 | 53.8 |
| 2G77 | 8.52 | 18 | 88 |  | 21.1 | 56.0 | 60.6 |  | 21.1 | 40.7 | 24.8 |  | 8.52 | 23.1 | 18.7 |  | 8.52 | 18.1 | 18.7 |  | 8.52 | 18.1 | 13.3 |
| 2HLE | 14.8 | 13 | 68 |  | 60.0 | 57.3 | 63.1 |  | 15.8 | 48.7 | 29.4 |  | 14.8 | 46.0 | 29.4 |  | 14.8 | 19.1 | 20.1 |  | 14.8 | 18.5 | 15.3 |
| 2HQS | 5.75 | 45 | 41 |  | 28.4 | 75.6 | 35.9 |  | 24.4 | 24.4 | 23.5 |  | 11.2 | 22.5 | 13.8 |  | 11.2 | 12.1 | 7.82 |  | 11.2 | 7.11 | 7.82 |
| 2OOB | 6.19 | 88 | 60 |  | 36.0 | 43.9 | 46.7 |  | 26.9 | 35.4 | 13.8 |  | 11.8 | 9.7 | 9.12 |  | 9.9 | 9.7 | 9.12 |  | 9.74 | 9.7 | 9.12 |
| 2OOR | 15.5 | 7 | 100 |  | 70.9 | 61.4 | 50.4 |  | 20.7 | 20.4 | 43.8 |  | 20.7 | 20.4 | 33.1 |  | 18.1 | 15.5 | 32.7 |  | 18.1 | 15.5 | 15.5 |
| 2VDB | 18.2 | 12 | 42 |  | 48.1 | 76.1 | 65.1 |  | 38.6 | 60.1 | 33.0 |  | 26.8 | 37.3 | 26.0 |  | 20.3 | 27.4 | 19.0 |  | 20.3 | 18.2 | 19.0 |
| 3BP8 | 26.3 | 4 | 33 |  | 79.3 | 78.3 | 43.0 |  | 45.4 | 72.6 | 38.1 |  | 41.9 | 72.6 | 38.1 |  | 40.1 | 41.4 | 37.7 |  | 36.4 | 41.4 | 35.2 |
| 3D5S | 13.8 | 7 | 50 |  | 40.3 | 39.0 | 48.9 |  | 26.7 | 39.0 | 31.2 |  | 24.8 | 29.5 | 30.9 |  | 22.1 | 24.2 | 18.0 |  | 17.5 | 19.0 | 18.0 |
|  |  |  |  |  |  |  |  |  |  |  |  |  |  |  |  |  |  |  |  |  |  |  |  |
| 1BGX | 27.6 | 47 | 21 |  | 67.3 | 78.6 | 57.5 |  | 54.3 | 52.0 | 47.6 |  | 27.6 | 37.3 | 36.7 |  | 27.6 | 37.3 | 36.7 |  | 27.6 | 31.3 | 27.6 |
| 1ACB | 4.44 | 68 | 100 |  | 21.1 | 32.7 | 22.0 |  | 12.3 | 14.9 | 14.4 |  | 12.3 | 6.92 | 8.95 |  | 12.3 | 4.44 | 8.95 |  | 9.28 | 4.44 | 8.95 |
| 1IJK | 6.33 | 50 | 90 |  | 28.1 | 42.9 | 25.2 |  | 25.2 | 15.3 | 17.5 |  | 15.2 | 6.88 | 6.88 |  | 15.2 | 6.88 | 6.88 |  | 6.88 | 6.88 | 6.88 |
| 1JIW | 3.08 | 62 | 100 |  | 16.0 | 25.1 | 48.8 |  | 11.1 | 25.1 | 18.3 |  | 11.1 | 21.0 | 14.8 |  | 11.1 | 9.49 | 12.0 |  | 9.6 | 9.49 | 12.0 |
| 1KKL | 4.61 | 44 | 60 |  | 32.1 | 73.3 | 11.6 |  | 14.3 | 29.3 | 8.88 |  | 14.3 | 21.4 | 8.88 |  | 12.1 | 17.8 | 8.88 |  | 9.2 | 12.6 | 8.88 |
| 1M10 | 9.18 | 18 | 34 |  | 60.4 | 62.0 | 65.1 |  | 16.6 | 52.8 | 16.6 |  | 16.6 | 21.8 | 16.6 |  | 16.6 | 12.8 | 9.18 |  | 9.18 | 9.18 | 9.18 |
| 1NW9 | 1.54 | 90 | 57 |  | 23.8 | 16.9 | 22.7 |  | 13.3 | 6.69 | 19.5 |  | 5.49 | 6.69 | 12.0 |  | 5.49 | 6.69 | 11.9 |  | 5.49 | 6.69 | 7.15 |
| 4CPA | 3.54 | 92 | 100 |  | 9.9 | 16.1 | 15.4 |  | 6.58 | 16.1 | 12.0 |  | 6.58 | 8.50 | 5.92 |  | 6.58 | 8.50 | 5.92 |  | 6.58 | 8.50 | 5.92 |
| 1GP2 | 19.7 | 100 | 100 |  | 38.8 | 48.8 | 41.5 |  | 31.7 | 30.0 | 23.6 |  | 19.7 | 23.6 | 23.6 |  | 19.7 | 19.7 | 19.7 |  | 19.7 | 19.7 | 19.7 |
| 1GRN | 5.87 | 57 | 13 |  | 31.0 | 53.6 | 50.2 |  | 22.4 | 33.5 | 31.7 |  | 9.4 | 23.8 | 23.8 |  | 9.4 | 9.4 | 23.8 |  | 9.4 | 8.57 | 21.6 |
| 1HE8 | 6.63 | 25 | 80 |  | 81.3 | 41.1 | 100 |  | 75.5 | 20.9 | 56.5 |  | 20.2 | 6.63 | 18.9 |  | 11.5 | 6.63 | 14.5 |  | 6.95 | 6.63 | 8.71 |
| 1I2M | 15.2 | 46 | 91 |  | 32.1 | 34.0 | 35.0 |  | 27.5 | 25.0 | 25.5 |  | 19.3 | 15.2 | 24.3 |  | 19.3 | 15.2 | 24.3 |  | 15.2 | 15.2 | 22.2 |
| 1IB1 | 12.9 | 40 | 32 |  | 39.9 | 39.3 | 29.1 |  | 27.5 | 26.1 | 23.4 |  | 19.2 | 19.4 | 19.4 |  | 17.8 | 17.2 | 19.4 |  | 16.6 | 15.1 | 17.5 |
| 1K5D | 20.3 | 19 | 16 |  | 76.3 | 71.5 | 30.4 |  | 34.0 | 32.3 | 30.4 |  | 25.4 | 25.4 | 30.4 |  | 25.4 | 25.4 | 20.3 |  | 20.3 | 20.3 | 20.3 |
| 1LFD | 5.64 | 100 | 100 |  | 35.9 | 18.4 | 25.2 |  | 11.9 | 18.4 | 15.3 |  | 11.4 | 16.5 | 9.34 |  | 11.2 | 5.64 | 9.34 |  | 11.2 | 5.64 | 8.99 |
| 1MQ8 | 44.3 | 3 | 100 |  | 58.4 | 70.9 | 60.0 |  | 53.3 | 57.3 | 56.2 |  | 48.0 | 55.4 | 51.5 |  | 48.0 | 51.5 | 50.2 |  | 48.0 | 48.6 | 48.6 |
| 1N2C | 11.6 | 45 | 45 |  | 18.2 | 100 | 47.7 |  | 11.6 | 18.2 | 41.7 |  | 11.6 | 11.6 | 11.6 |  | 11.6 | 11.6 | 11.6 |  | 11.6 | 11.6 | 11.6 |
| 1R6Q | 12.1 | 38 | 100 |  | 28.3 | 48.1 | 54.0 |  | 26.6 | 42.4 | 23.5 |  | 12.1 | 35.0 | 23.5 |  | 12.1 | 23.8 | 23.5 |  | 12.1 | 20.2 | 18.3 |
| 1SYX | 3.31 | 85 | 88 |  | 31.4 | 49.0 | 35.4 |  | 6.27 | 30.7 | 32.8 |  | 6.27 | 20.4 | 18.3 |  | 6.27 | 15.4 | 16.7 |  | 6.27 | 13.2 | 14.8 |
| 1WQ1 | 11.8 | 14 | 12 |  | 49.3 | 50.6 | 25.5 |  | 12.9 | 21.8 | 18.1 |  | 12.9 | 19.2 | 18.1 |  | 12.9 | 12.9 | 18.1 |  | 12.9 | 12.9 | 18.1 |
| 1XQS | 14.1 | 14 | 11 |  | 54.8 | 55.5 | 66.6 |  | 27.5 | 37.1 | 38.1 |  | 24.1 | 29.2 | 29.2 |  | 15.9 | 14.1 | 29.2 |  | 14.1 | 14.1 | 29.2 |
| 2AYO | 4.22 | 40 | 63 |  | 13.9 | 52.2 | 53.5 |  | 9.2 | 13.1 | 30.6 |  | 4.24 | 13.1 | 14.9 |  | 4.24 | 13.1 | 9.2 |  | 4.24 | 11.4 | 9.2 |
| 2CFH | 1.53 | 84 | 75 |  | 3.54 | 32.0 | 39.4 |  | 1.53 | 23.7 | 9.1 |  | 1.53 | 5.89 | 6.72 |  | 1.53 | 5.89 | 6.72 |  | 1.53 | 5.89 | 6.72 |
| 2H7V | 2.17 | 100 | 100 |  | 70.9 | 9.23 | 100 |  | 56.7 | 3.74 | 11.9 |  | 8.25 | 3.74 | 5.43 |  | 8.25 | 3.57 | 5.43 |  | 2.22 | 3.53 | 5.36 |
| 2HRK | 2.66 | 100 | 76 |  | 49.2 | 48.3 | 52.0 |  | 35.4 | 17.0 | 25.1 |  | 22.5 | 17.0 | 17.0 |  | 21.6 | 14.5 | 15.1 |  | 9.1 | 8.94 | 9.1 |
| 2J7P | 15.0 | 30 | 27 |  | 58.1 | 37.0 | 64.3 |  | 21.5 | 15.0 | 23.5 |  | 21.5 | 15.0 | 23.5 |  | 15.3 | 15.0 | 16.3 |  | 15.3 | 15.0 | 16.3 |
| 2NZ8 | 14.8 | 12 | 80 |  | 35.0 | 60.9 | 54.7 |  | 18.2 | 57.8 | 51.7 |  | 18.2 | 26.8 | 17.7 |  | 16.9 | 21.3 | 17.7 |  | 16.9 | 21.1 | 15.6 |
| 2OZA | 8.98 | 36 | 33 |  | 16.9 | 41.3 | 69.5 |  | 16.9 | 8.98 | 33.7 |  | 11.6 | 8.98 | 29.6 |  | 11.6 | 8.98 | 17.2 |  | 11.6 | 8.98 | 8.98 |
| 2Z0E | 9.3 | 12 | 89 |  | 60.2 | 58.6 | 57.5 |  | 34.8 | 45.7 | 34.7 |  | 15.0 | 36.7 | 9.5 |  | 15.0 | 17.1 | 9.5 |  | 15.0 | 13.5 | 9.5 |
| 3CPH | 49.8 | 13 | 100 |  | 70.4 | 64.2 | 65.9 |  | 55.6 | 55.6 | 65.6 |  | 52.0 | 49.8 | 58.6 |  | 51.0 | 49.8 | 56.7 |  | 49.8 | 49.8 | 55.6 |
|  |  |  |  |  |  |  |  |  |  |  |  |  |  |  |  |  |  |  |  |  |  |  |  |
| 1E4K | 29.5 | 10 | 2 |  | 42.2 | 96.1 | 57.2 |  | 42.2 | 44.1 | 52.8 |  | 38.6 | 44.1 | 43.9 |  | 32.6 | 44.1 | 43.9 |  | 32.6 | 44.1 | 33.3 |
| 2HMI | 18.7 | 100 | 89 |  | 36.7 | 21.8 | 26.7 |  | 34.3 | 18.7 | 21.8 |  | 18.7 | 18.7 | 18.7 |  | 18.7 | 18.7 | 18.7 |  | 18.7 | 18.7 | 18.7 |
| 1F6M | 7.04 | 22 | 100 |  | 50.7 | 49.5 | 54.8 |  | 26.1 | 22.4 | 49.1 |  | 13.5 | 7.04 | 14.9 |  | 13.5 | 7.04 | 14.9 |  | 11.1 | 7.04 | 13.7 |
| 1FQ1 | 8.88 | 88 | 80 |  | 62.4 | 60.3 | 63.0 |  | 40.4 | 51.2 | 43.9 |  | 28.0 | 19.5 | 25.4 |  | 21.6 | 19.5 | 23.0 |  | 14.5 | 19.5 | 22.0 |
| 1PXV | 6.76 | 62 | 36 |  | 41.1 | 27.0 | 29.9 |  | 24.1 | 22.0 | 25.9 |  | 15.4 | 18.2 | 16.8 |  | 12.0 | 16.3 | 16.5 |  | 12.0 | 11.1 | 6.76 |
| 1ZLI | 18.2 | 14 | 46 |  | 52.7 | 43.4 | 49.0 |  | 25.2 | 24.0 | 30.3 |  | 19.1 | 23.8 | 27.8 |  | 18.2 | 23.8 | 26.7 |  | 18.2 | 21.3 | 25.7 |
| 2O3B | 8.16 | 50 | 50 |  | 48.9 | 17.5 | 52.4 |  | 18.3 | 17.5 | 37.2 |  | 16.9 | 8.16 | 23.3 |  | 14.4 | 8.16 | 17.1 |  | 8.16 | 8.16 | 14.8 |
| 1ATN | 22.0 | 90 | 83 |  | 46.0 | 77.5 | 42.9 |  | 46.0 | 31.7 | 25.1 |  | 27.0 | 23.2 | 22.0 |  | 22.0 | 23.2 | 22.0 |  | 22.0 | 22.0 | 22.0 |
| 1BKD | 6.74 | 23 | 65 |  | 24.4 | 60.7 | 65.9 |  | 13.1 | 19.0 | 24.4 |  | 13.1 | 16.5 | 24.4 |  | 9.94 | 9.94 | 21.7 |  | 6.74 | 9.94 | 13.1 |
| 1DE4 | 19.8 | 94 | 77 |  | 57.7 | 62.6 | 70.7 |  | 43.7 | 51.9 | 66.6 |  | 19.8 | 19.8 | 37.6 |  | 19.8 | 19.8 | 29.4 |  | 19.8 | 19.8 | 19.8 |
| 1EER | 9.3 | 34 | 32 |  | 26.3 | 56.6 | 13.4 |  | 26.3 | 11.5 | 13.4 |  | 11.6 | 9.3 | 13.4 |  | 11.6 | 9.3 | 13.4 |  | 9.3 | 9.3 | 13.4 |
| 1FAK | 11.8 | 100 | 100 |  | 50.5 | 35.5 | 91.1 |  | 27.6 | 33.3 | 45.5 |  | 22.1 | 24.8 | 24.7 |  | 19.7 | 20.0 | 22.4 |  | 19.7 | 20.0 | 22.4 |
| 1H1V | 42.7 | 17 | 0 |  | 83.7 | 56.7 | 64.2 |  | 63.5 | 56.7 | 45.8 |  | 49.5 | 50.7 | 42.7 |  | 47.0 | 47.8 | 42.7 |  | 42.7 | 42.7 | 42.7 |
| 1IBR | 13.2 | 59 | 53 |  | 50.9 | 71.4 | 85.7 |  | 34.7 | 39.7 | 51.9 |  | 13.2 | 37.2 | 33.9 |  | 13.2 | 37.2 | 33.9 |  | 13.2 | 32.3 | 30.9 |
| 1IRA | 22.5 | 40 | 41 |  | 37.8 | 38.0 | 58.2 |  | 24.7 | 32.2 | 30.8 |  | 24.7 | 25.6 | 27.3 |  | 24.7 | 25.6 | 27.3 |  | 24.7 | 25.6 | 25.9 |
| 1JK9 | 14.9 | 17 | 64 |  | 35.0 | 53.4 | 30.3 |  | 26.8 | 40.8 | 30.3 |  | 23.4 | 14.9 | 26.7 |  | 23.4 | 14.9 | 26.7 |  | 20.0 | 14.9 | 14.9 |
| 1JMO | 18.1 | 8 | 62 |  | 81.9 | 49.6 | 50.5 |  | 55.7 | 31.1 | 49.1 |  | 25.7 | 30.8 | 20.7 |  | 18.1 | 18.1 | 20.7 |  | 18.1 | 18.1 | 18.1 |
| 1JZD | 4.00 | 33 | 57 |  | 38.4 | 24.1 | 9.44 |  | 17.7 | 14.7 | 9.44 |  | 17.7 | 14.4 | 9.44 |  | 14.8 | 14.4 | 9.44 |  | 11.1 | 14.4 | 6.29 |
| 1R8S | 8.55 | 64 | 56 |  | 64.8 | 57.3 | 44.8 |  | 19.7 | 18.1 | 19.7 |  | 19.7 | 16.9 | 17.9 |  | 19.7 | 15.4 | 14.0 |  | 13.1 | 8.55 | 14.0 |
| 1Y64 | 18.7 | 96 | 12 |  | 49.6 | 81.7 | 69.4 |  | 25.5 | 56.9 | 39.5 |  | 18.7 | 18.7 | 34.1 |  | 18.7 | 18.7 | 34.1 |  | 18.7 | 18.7 | 18.7 |
| 1ZM4 | 6.24 | 41 | 90 |  | 31.9 | 68.1 | 41.4 |  | 31.4 | 29.3 | 29.0 |  | 27.0 | 28.8 | 18.4 |  | 12.2 | 13.2 | 13.2 |  | 12.2 | 13.2 | 9.4 |
| 2C0L | 15.3 | 63 | 14 |  | 27.9 | 25.5 | 54.6 |  | 19.6 | 21.9 | 21.2 |  | 18.1 | 19.3 | 19.4 |  | 18.1 | 18.1 | 15.3 |  | 17.1 | 18.1 | 15.3 |
| 2I9B | 4.62 | 10 | 57 |  | 30.6 | 55.1 | 69.8 |  | 28.5 | 47.5 | 30.3 |  | 25.0 | 34.9 | 27.3 |  | 8.52 | 13.6 | 15.4 |  | 8.52 | 13.6 | 13.9 |
| 2IDO | 5.76 | 50 | 57 |  | 30.8 | 52.8 | 18.9 |  | 17.2 | 14.1 | 9.34 |  | 17.2 | 14.1 | 8.47 |  | 7.57 | 9.34 | 8.47 |  | 7.57 | 9.34 | 8.47 |
| 2OT3 | 28.6 | 7 | 75 |  | 54.7 | 46.3 | 57.2 |  | 52.6 | 39.5 | 55.6 |  | 34.5 | 30.3 | 44.2 |  | 33.3 | 30.3 | 37.1 |  | 28.6 | 28.8 | 28.6 |
